# Recent advances in understanding/assessing toxicity to the epigenome

**DOI:** 10.12688/f1000research.9649.1

**Published:** 2017-02-01

**Authors:** Kevin Sweder

**Affiliations:** 1 Forensic and National Security Sciences Institute, Center for Science & Technology, Syracuse University, Syracuse, NY, USA

**Keywords:** genetic toxicology, non-genotoxic agents, epigenetic mechanisms

## Abstract

The ability of non-genotoxic agents to induce cancer has been documented and clearly requires a reassessment of testing for environmental and human safety. Drug safety testing has historically relied on test batteries designed to detect DNA damage leading to mutation and cancer. The standard genetic toxicology testing battery has been a reliable tool set to identify small molecules/chemicals as hazards that could lead to genetic changes in organisms and induction of cancer. While pharmaceutical companies and regulatory agencies have extensively used the standard battery, it is not suitable for compounds that may induce epigenetic changes. Additionally, many pharmaceutical companies have changed their product portfolios to include peptides and/or other biological molecules, which are not expected to be genotoxic in their own right. If we are to best use our growing knowledge regarding chemicals and biomolecules that induce heritable changes via epigenetic mechanisms, then we must ask what changes may be needed in our testing paradigm to predict long-term downstream effects through epigenetic mechanisms.

## Introduction

As more is learned about chemicals and biomolecules that induce heritable changes via epigenetic mechanisms, we must ask what changes may be needed in our testing paradigm to predict risk from long-term downstream effects and transgenerational effects. What has become apparent in recent years is the ability of non-genotoxic agents to cause cancer. These non-genotoxic agents do not directly damage DNA in exposed cells. Rather, they may induce expression or utilization of non-coding RNAs and/or modify DNA or proteins in chromatin without altering the primary DNA base sequence. In light of the limits of standard genetic toxicity testing strategies for identifying epigenetic hazards, the goal of this review is to identify and discuss opportunities/challenges in developing products that have an epigenetic mode of action as well as new techniques for identifying epigenetic hazards which may complement or diverge from standard testing. Additionally, we will discuss and suggest how toxicities could be put into perspective for filing with regulatory agencies, including which new tests would be required to suitably address safety concerns for epigenetic drugs and compounds in humans as well as means for assessing their efficacy.

## What is epigenetics?

**Figure 1.**
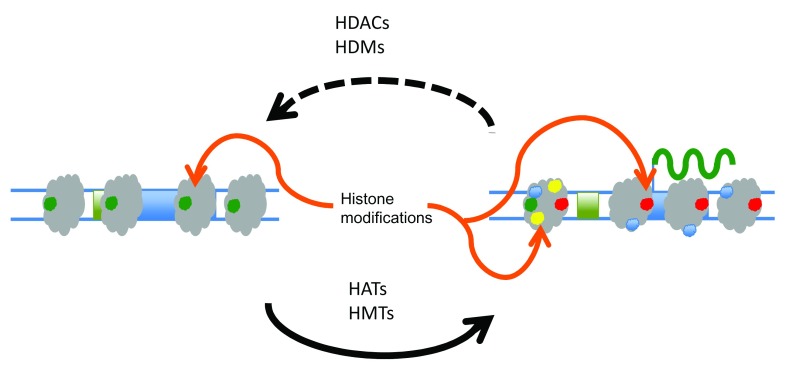
Epigenetic changes in eukaryotic cells. Accessibility of information in the eukaryotic genome may be controlled through epigenetic modifications. For example, increases or decreases in the transcription of genes may result from a delicate balance of enzymatic activity that can add or remove acetyl or methyl groups on histones. During cell growth and division, DNA is replicated and most epigenetic modifications are transiently lost or removed (dashed line). Cellular processes exist which re-establish epigenetic modifications to DNA and associated proteins (solid lines). HAT, histone acetyltransferase; HDAC, histone deacetylase; HDM, histone demethylase; HMT, histone methyltransferase.

What is meant by epigenetic changes to eukaryotic chromatin? In this review, epigenetics are those changes or modifications to DNA or the proteins associated with DNA and non-coding RNAs that are inherited in a non-Mendelian fashion. The modifications include methylation, acetylation, ubiquitylation, phosphorylation, sumoylation, and other chemical moieties covalently bound to DNA or chromosomal (or chromosome-associated) proteins. These modifications alter function but do not necessarily alter the genetic sequence within DNA; rather, they alter the access of biochemical machinery to that information. During cell growth and division, DNA is replicated and most epigenetic modifications are transiently removed. Cellular processes exist which re-establish epigenetic modifications to DNA and associated proteins ^1, 2^. As shown in Figure 1 for core histone modifications, dedicated enzymatic pathways exist for the addition of acetyl or methyl groups onto lysine and/or arginine moieties (e.g. histone acetyltransferases [HATs] and histone methyltransferases [HMTs]) within histones and for their removal (e.g. histone deacetylases [HDACs] and histone demethylases [HDMs]).

An analogy that I find helps non-scientists to understand epigenetics is a comparison of the human genome to a cookbook. The information contained on the pages of the cookbook is the DNA sequence or genetic information. If there is a favorite recipe within that cookbook to which a cook must frequently refer, he or she should not have to thumb through a large portion of the cookbook to find that recipe each time. If the cook puts a sticky note or flag on the page containing the recipe, then he or she would be able to find that recipe much more easily. Similarly, an unpopular recipe can be made less accessible by placing a paperclip on the pages containing the recipe. In this analogy, the sticky notes or paperclips represent the epigenetic changes that alter accessibility without changing the text (DNA base sequence) on the pages of the cookbook. What is the effect of these non-coding RNAs or modifications to DNA and proteins to cellular and organismal function? Many groups have demonstrated that epigenetic changes in promoter regions of genes are correlated with increased or decreased transcription of those genes ( 2 and several references therein). Conversely, epigenetic changes within genes appear to be correlated with changes in transcription ( 3 and some references therein). Some of these epigenetic changes, such as DNA methylation at CpG dinucleotides, may be very long-lived, lasting through many cell generations. Thus, exposure to exogenous agents may result in long-lived changes in somatic cells and tissues. It may be that these long-lived changes (multiple cell generations) in epigenetic profiles and corresponding transcriptional changes may represent the mechanism whereby non-genotoxic agents cause neoplastic transformation and cancer. However, to best understand the mechanisms of epigenetic modifications, there is a critical need for research to determine which epigenetic changes cause the observed altered phenotypes and which epigenetic changes are, in turn, results of that phenotypic change, i.e. which are causative and which are resultant.

## Clinical experience with epigenetic drugs

There are several drugs approved by the FDA that act through epigenetic mechanisms and for which clinical drug safety testing exists, e.g. inhibitors of HDACs and HATs. While both HDACs and HATs are targets for drug development, most of the epigenetic drugs that have made it into clinical trials are HDAC inhibitors ^4^. For HDAC inhibitors, like Vorinostat (Merck), Romidepsin (Celgene), F448, and Panobinostat (Novartis), some of the toxicological outcomes include nausea, diarrhea, and vomiting. More deleterious outcomes of HDAC inhibitors include thrombocytopenia, leukopenia, and other anemias, as well as central nervous system effects. On the nucleic acid side, in clinical trials, inhibitors of DNA methyltransferases (e.g. azacitidine [5-azacytidine; Vidaza and Celgene] and decitabine [5-aza-2 '-deoxycytidine; Dacogen and SuperGen]) were found to be associated with nausea, vomiting, diarrhea, constipation, anorexia, neutropenia, thrombocytopenia, liver function abnormalities, and renal failure in patients during sepsis and hypotension ^5– 7^.

While the aim of genetic toxicological studies has focused on the ability of compounds to induce mutations and/or cancer in humans ^8^, a critical aspect of epigenetic changes includes germ cell effects and thus transgenerational phenotypic changes. Evidence from epidemiological studies indicates that epigenetic changes induced by environmental factors such as starvation or stress are associated with transcriptional and phenotypic changes in the exposed population, their children, and their grandchildren. Studies of children and grandchildren of parents who lived through a famine in Sweden in the 1850s indicate that transcriptional changes were passed from the parents to their children and grandchildren ^9^. Such transgenerational effects might be mediated by epigenetic mechanisms. Similarly, Dutch women undergoing starvation during mid-pregnancy in World War II subsequently had children who delivered undersized grandchildren ^10^. Holocaust survivors, their children, and their grandchildren led the authors to a similar conclusion that extreme stress induced epigenetic changes that were inherited in a non-Mendelian manner ^11^. Work published by Michael Skinner and colleagues showed that rats exposed to vinclozolin, a fungicide, caused changes in DNA methylation different from the control group and these methylation changes presumably caused a developmental effect in subsequent generations of rats ^12^.

A key element in any drug safety assessment is the transferability of an assay from laboratory to laboratory. Efforts to repeat the observation of transgenerational effects have been mixed. A critical question to address is the similarity or lack thereof in experimental design and statistical analysis. Another study by Schneider *et al*. tried to replicate the experiments of Anway *et al*. using vinclozolin in rats ^13^. Their results led them to conclude that any transgenerational effects were not significantly different from control groups. There are several differences between the two studies that might explain some of the disparity in conclusions. Of note, in their study, Schneider and co-workers used different rat strains than those used by Anway *et al*. Perhaps more importantly, rats were dosed orally in the Schneider study, whereas Anway dosed the rats intraperitoneally ^12, 13^. Thus, in Anway’s study, vinclozolin was not undergoing first-pass metabolism in exposed rats, leading to a different exposure profile than that of rats in the study by Schneider *et al*. In the study mentioned above, Anway *et al*. performed a two-way analysis of variance (ANOVA) to determine the significance of transgenerational effects. The statistical analysis used in Schneider *et al*. was a one-way ANOVA with Kruskal-Wallis correction, which is a comparison of the mean ranks instead of a test of equality of means. Finally, a single dose was used to dose rats in the study by Anway *et al*., while Schneider *et al*. used two groups of rats each administered a different dose. This last point is important because the levels at which transgenerational effects were observed are manifold greater than the no observable adverse effect level (NOAEL) or lowest observable adverse effect level (LOAEL).

## Predicting apical endpoints based on epigenetic changes

With the establishment of pathological and transgenerational effects following exposure to epigenetic compounds, several labs have tried to identify which of the genetic changes are responsible for these adverse outcomes. In one study, rats were exposed to nine genotoxic and non-genotoxic compounds, and cells were then harvested for DNA methylation assays ^14^. The authors chose to examine DNA methylation at the genomic level rather than interrogating particular genes and their promoters. By comparing the DNA methylation observed in genotoxic-treated animals versus non-genotoxic-treated animals, they identified the DNA methylation sites that were differentially modified following exposure to non-genotoxic, epigenetic-inducing compounds.

Additional considerations related to investigations of genome-wide association studies (GWAS) and/or epigenome-wide association studies (EWAS) include single-dose versus multiple-dose studies and longitudinal versus transverse studies. Lee *et al*. performed a GWAS to compare a single dose with multiple doses of known genotoxic and non-genotoxic compounds in rats. Additionally, they examined expression following the administration of a single dose and multiple doses of four test compounds. The expression levels following single and multiple doses for genotoxic and non-genotoxic compounds (and controls) were not distinguishable by principal component analysis (PCA). The only exception was expression changes following multiple treatment with DL-ethionine ^3^. The authors then offered the explanation that, within the total genomic expression that is independent of treatment protocol, the expression changes within a few genes cannot be discerned. Thus, criteria were imposed to enable the comparison of differentially expressed genes among the various treatment conditions.

Comparison of genes whose expression varied significantly from control animals enabled discrimination between single doses of genotoxic and non-genotoxic compounds. Furthermore, all four of the test agents clustered with the genotoxic compounds. The authors postulated that dosing with any of the compounds used is sufficient to initiate a cellular response that could diminish the expression changes that are unique to the particular compounds. Hierarchical clustering of expression changes of genes after single doses lacked the consistency to ascribe a mechanistic pathway for the different clusters.

In contrast to the results noted in the preceding paragraph, gene expression changes following multiple dosing of the four test agents varied significantly from control animals and were most similar to expression changes induced by non-genotoxic compounds. Also, the additional time for multiple dosing resulted in gene expression changes returning back to normal for the test compounds and the non-genotoxic agents. In contrast, genotoxic agents induced discernible changes as time passed. Importantly, clustering of differentially expressed genes for genotoxic agents enabled the identification of specific biochemical pathways, e.g. genes encoding proteins in the p53 DNA damage response pathway, which appeared indicative of exposure to genotoxic agents.

## Future directions

Variations in methods, results, and interpretations indicate that the field of assessing epigenetic alterations requires a consortium of laboratories performing the same experiments and using the same protocols and the same statistical analysis of the data to demonstrate the transferability of this method for use and drug safety tests.

Several considerations to eliminate the variability in study-to-study and laboratory-to-laboratory comparison of data have been clearly described by Birney *et al*. ^15^. Determination of epigenetic changes should not be averaged over mixed populations of cells. Longitudinal studies of epigenetic changes in individual animals could indicate which epigenetic changes are responsible for phenotypic changes in individual animals and their progeny.

Given the evidence that non-genotoxic compounds acting through epigenetic mechanisms are associated with elevated incidence of cancer, how can we incorporate this mechanistic information into drug safety studies? Given that most of the experiments that demonstrate transgenerational transmission of phenotypic changes are by necessity long-term animal studies, what can be done in the short term to inform drug safety assessment? Are there tractable model systems to demonstrate heritable epigenetic changes? One possible solution is to use small non-mammalian organisms such as roundworms ( *Caenorhabditis elegans*) or zebrafish ( *Danio rerio*) for the investigation of developmental and transgenerational effects.

*C. elegans* has many attributes that make it an excellent organism in which to study epigenetics ^16^. *C. elegans* is a free-living nematode with defined cell lineages and many proteins sharing homology to human proteins. Many developmental decisions in *C. elegans* are mediated through epigenetic mechanisms. Additionally, *C. elegans* has a transparent body that makes microscopic imaging of tissues and labeled proteins much easier than in mammals. For example, *C. elegans* expressing GFP-tagged neuronal populations (pan, dopaminergic, or GABAergic) were exposed to the herbicide glyphosate and the effect upon specific populations of neurons was determined ^17^. In this study, dopaminergic neurons were more sensitive than GABAergic neurons to glyphosate exposure. Thus, dopaminergic degradation might serve as an early indicator of neuronal vulnerability to glyphosate exposure. It remains to be determined what type of epigenetic modification is responsible for neuronal effects.

The zebrafish is another organism that might prove useful for screening epigenetic compounds for toxicities and transgenerational effects. Zebrafish are tropical freshwater fish that typically grow to 2–4 cm in length ^18^. They are transparent from fertilized eggs through >72 hours. Being chordates, they share similar biochemical processes and homology among the proteins performing these processes, e.g. DNA replication and chromatin structure. Like *C. elegans*, zebrafish are relatively inexpensive to maintain. Finally, because they lay eggs, maternal exposure to epigenetic compounds does not affect the F2 generation directly, so zebrafish are especially useful for investigating heritable epigenetic changes.

Finally, observation of phenotypic changes in model organisms exposed to epigenetic compounds raises concern over drug or environmental safety for humans or other organisms. Such concerns are valid without knowing the mechanism whereby the adverse effect occurs. However, understanding the mechanism whereby the adverse effect takes place may help establish a screening assay that can predict which epigenetic compounds are carcinogenic and obviate the need for some animal studies.

Given that animal studies have indicated the suitability of using gene expression profiling to identify pathways could prove useful for the determination of carcinogenic mechanisms ^3^, a similar undertaking in model organisms, such as *C. elegans*, zebrafish, or cultured cells (e.g. TK6), seems warranted. Additionally, 6-month carcinogenicity studies in transgenic mice could identify hazardous epigenetic drugs and support risk assessment. Identifying a model system that avoids the use of larger animals while still providing determination of carcinogenic mechanisms would be a boon to industry and regulatory agencies alike. Once established, these model systems might enable the elucidation of the particular epigenetic changes responsible for gene expression changes involved in carcinogenesis.

Box 1. **Executive Summary**

- The standard genetic toxicology battery may not be sufficient to identify epigenomic toxicity and associated apical toxicities.
  - Are specific epigenetic modifications causing specific apical endpoints or are the epigenetic modifications a result of apical phenotype?
- FDA-approved epigenetic drugs have adverse toxicological effects at readily achieved exposures in clinical trials.
  - Cardiotoxicity and hematologic toxicities.
  - Is it likely that epigenetic drugs may be carcinogenic at therapeutic doses?
- What testing is likely to identify biomarkers of adverse effects that are potentially carcinogenic and/or transgenerational?
  - Genome-wide association studies (GWAS) and/or epigenome-wide association studies (EWAS).
  - Include single-dose versus multiple-dose studies.
  - Longitudinal versus transverse studies.
- Standardization of Approaches- Large inter-laboratory studies are needed to identify causal epigenetic modifications leading to adverse endpoints.
  - EWAS.
  - Such studies should incorporate multiple-dose, longitudinal studies.
- Until cause and effect of epigenetic drug exposure and adverse outcomes (like cancer) are demonstrated, reliance on phenotypic changes following exposure to epigenetic drugs are needed.
  - Determination of doses/exposures causing transgenerational effects versus the no observable adverse effect level (NOAEL) or lowest observable adverse effect level (LOAEL).
  - 6-month carcinogenicity studies in transgenic mice could identify hazardous epigenetic drugs and support risk assessment.
  - Model systems such as zebrafish and *C. elegans* may prove useful for identification of biomarkers of adverse endpoints and transgenerational inheritance.

## Abbreviations

ANOVA, analysis of variance; EWAS, epigenome-wide association study; GWAS, genome-wide association study; HAT, histone acetyltransferase; HDAC, histone deacetylase; HDM, histone demethylase; HMT, histone methyltransferase; LOAEL, lowest observable adverse effect level; NOAEL, no observable adverse effect level; PCA, principal component analysis.
